# Supplementary material for: SPADE: spatial deconvolution for domain specific cell-type estimation
Source: Commun Biol. 2024 Apr 17;7:469. doi: 10.1038/s42003-024-06172-y (PMC11024133; doi:10.1038/s42003-024-06172-y)
Supplement: Supplementary file 2 — Supplementary Information [file 42003_2024_6172_MOESM2_ESM.pdf]

SPADE: Spatial Deconvolution for Domain Specific Cell-type Estimation

Yingying Lu[1], Qin Chen[2], and Lingling An*[1,3,4]

[1]Interdisciplinary Program in Statistics and Data Science, University of Arizona, Tucson, AZ 85721, USA
[2]College of Pharmacy, University of Arizona, Tucson, AZ 85721, USA
[3]Department of Biosystems Engineering, University of Arizona, Tucson, AZ 85721, USA
[4]Department of Epidemiology and Biostatistics, University of Arizona, Tucson, AZ 85721, USA

Supplementary Information

Supplementary Figures

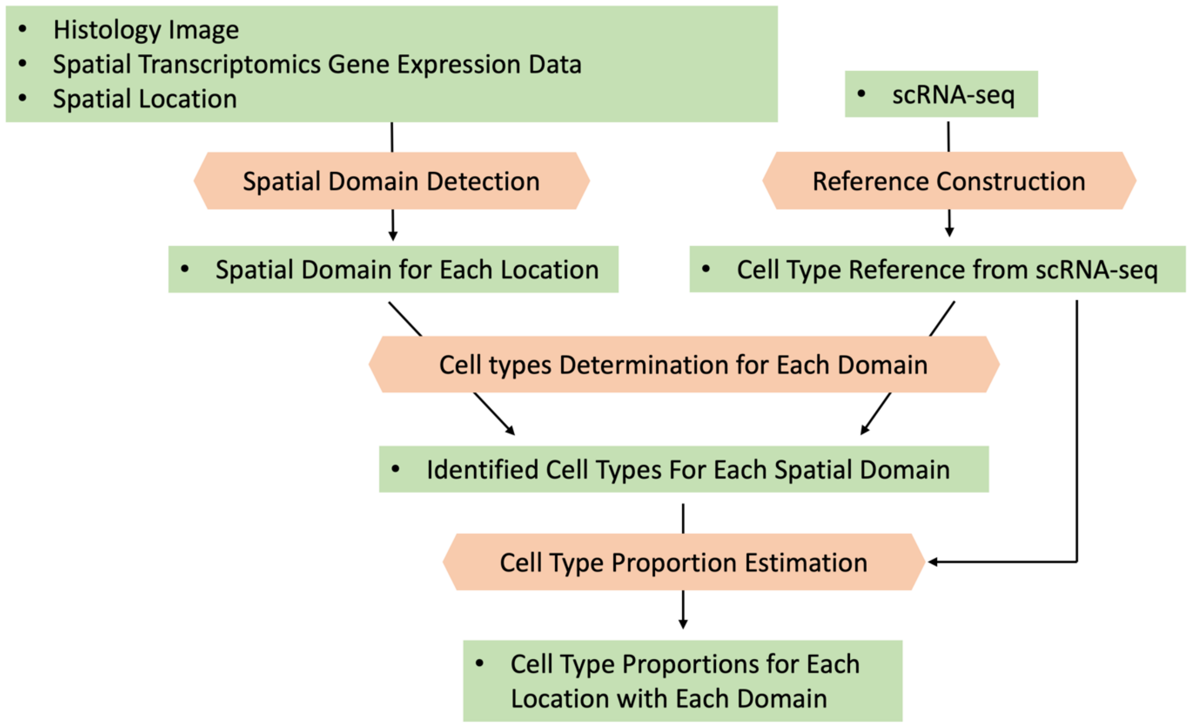

Supplementary Figure 1. Schematic overview of the SPADE process. Green square shapes represent data inputs and outputs, while orange diamond shapes denote the stages or steps of the analysis. The workflow begins with data inputs, which include a Histology Image, Spatial Transcriptomics Gene Expression Data, and Spatial Location information. These inputs feed into the first stage, 'Spatial Domain Detection,' where spatial domains are identified for each location. The output from this stage is 'Spatial Domain for Each Location,' indicating the categorization of spatial locations into domains. Concurrently, 'Reference Construction' is a stage that uses scRNA-seq data to create a cell type reference. The cell type reference, an output depicted as 'Cell Type Reference from scRNA-seq,' is then utilized in the 'Cell Types Determination for Each Domain' stage, leading to the identification of 'Identified Cell Types For Each Spatial Domain.' The final stage, 'Cell Type Proportion Estimation,' estimates the proportions of cell types across locations, culminating in the output 'Cell Type Proportions for Each Location within Each Domain,' which represents the distribution of cell types within the spatial framework of the domains.

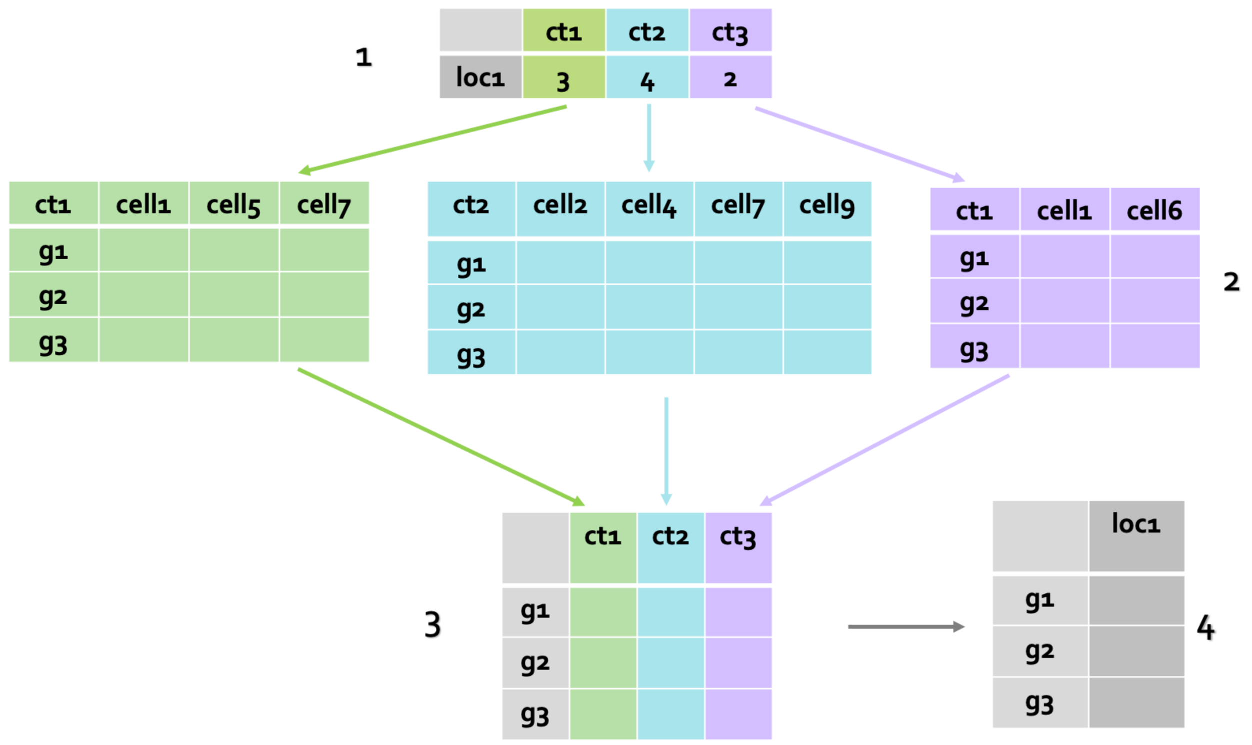

Supplementary Figure 2. Construction of synthetic spatial transcriptomics data. The spaGCN can be used to determine the spatial domains that contain varying numbers of unique locations. With the domain information, we can then construct synthetic spatial gene expression based on single-cell RNA-seq data and map the synthetic data to each location. To elaborate further, we first use the Dirichlet distribution to generate random proportions for each location. These proportions are then utilized as a ground truth for later method comparisons. For each domain, we pre-define one dominant cell type and several minor cell types. Based on the total number of cells for each cell type in the single-cell data, we convert the random proportion to random cell counts for each cell type. Second, we randomly select cells based on the cell counts from the previous step, from each cell type in the scRNA-seq data for each location. Third, we aggregate gene expression across all cells for each cell type to obtain cell type-specific gene expression for each location. Fourth, we summarize cell type-specific gene expression within each location to obtain location-specific gene expression. Ultimately, we obtain a gene-by-location matrix that is utilized as artificial spatial transcriptomics data.

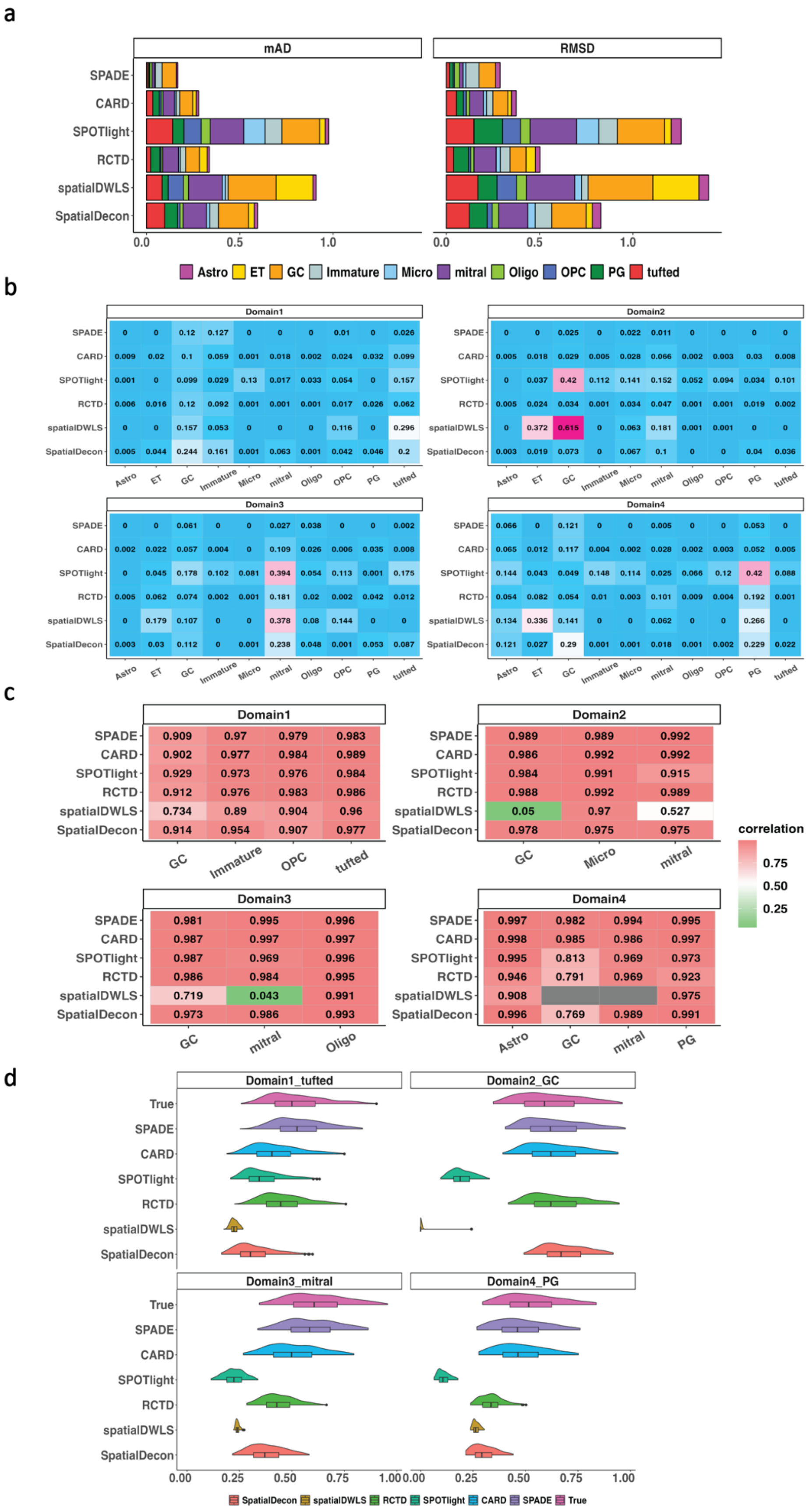

a
mAD
RMSD
SPADE
CARD
SPOTlight
RCTD
spatialDWLS
SpatialDecon
0.0
0.5
1.0
Astro
ET
GC
Immature
Micro
mitral
Oligo
OPC
PG
tufted
b
Domain1
Domain2
Domain3
Domain4
mAD
0.6
0.4
0.2
0.0
c
correlation
0.75
0.50
0.25
d
Domain1_tufted
Domain2_GC
Domain3_mitral
Domain4_PG
True
0.00
0.25
0.50
0.75
1.00

Supplementary Figure 3. Simulation results on the MOB data. a. The mean absolute deviance(mAD) and rooted mean square deviance(RMSD) between estimated proportion and true proportion for each cell type by different methods. Cell types are represented by distinct colors. b. Calculated mAD for each cell type within each spatial domain by different methods. Lower value is preferred. c. Correlation between estimated and true cell type proportion for each domain. Only cell type that truly presents in each domain will be listed in the figure. d. The proportion for the dominant cell type within each spatial domain. color represents different methods.

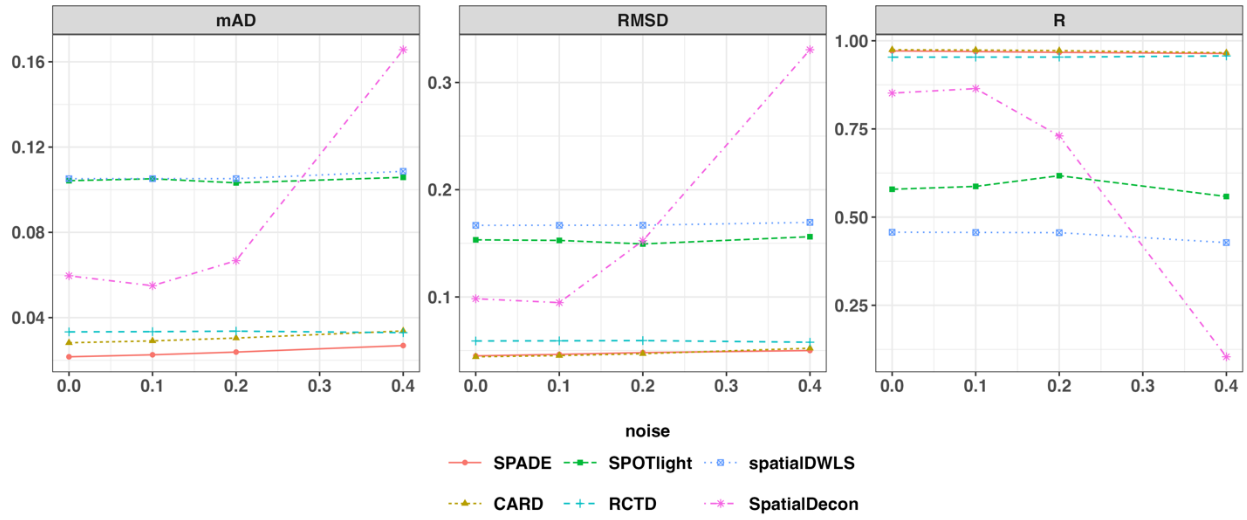

Supplementary Figure 4. Performance comparison at different noise levels in the MOB data. The x-axis represents noise levels, and the y-axis is the values corresponding to each evaluation metric. Each panel illustrates a distinct evaluation outcome. Results obtained from different methods are represented by colored lines. Lower values are desirable for metrics like mAD and RMSD, while a higher value is preferable for the correlation coefficient, denoted as R.

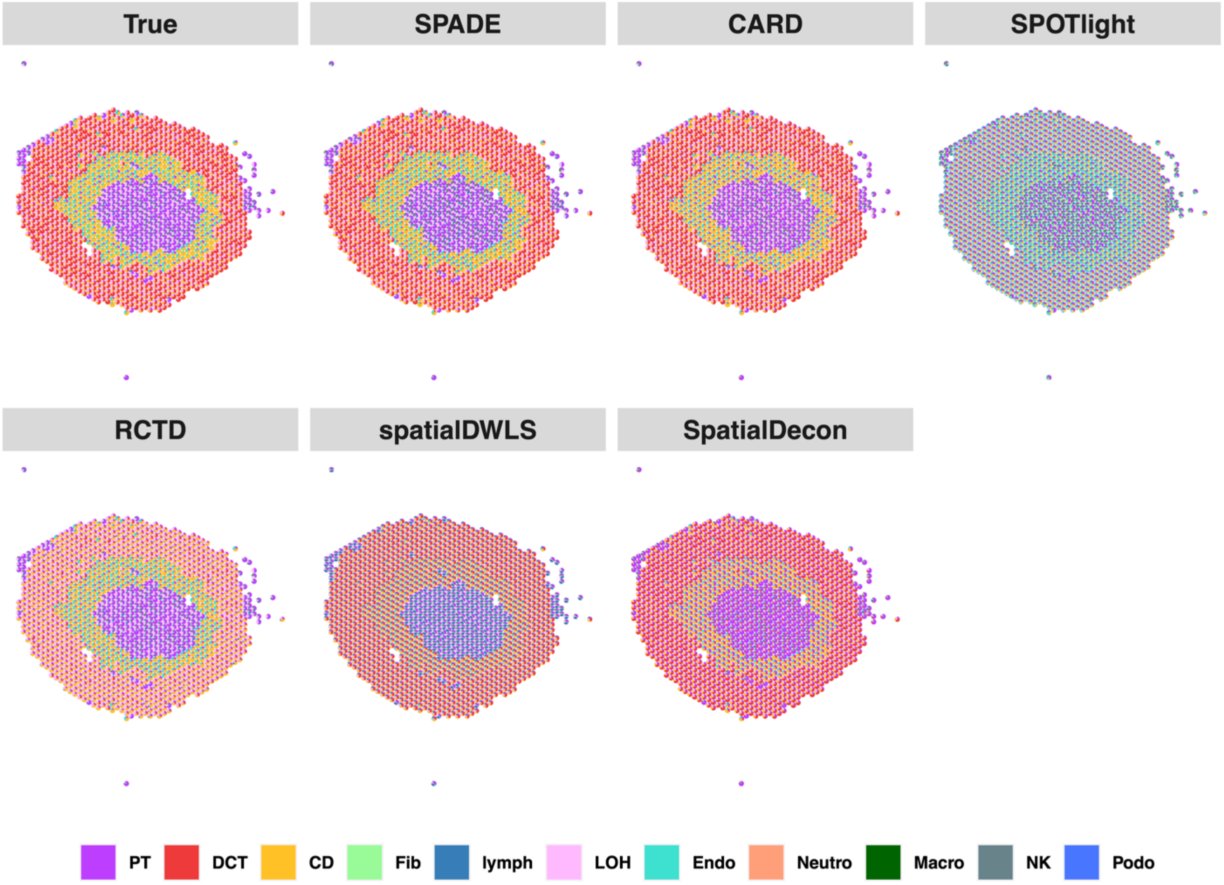

Supplementary Figure 5. Simulation results on the mouse kidney data. Scatter pie plot for predicted cell type proportions at each spatial location. Each location is denoted by a pie plot indicated by different composition of cell types.

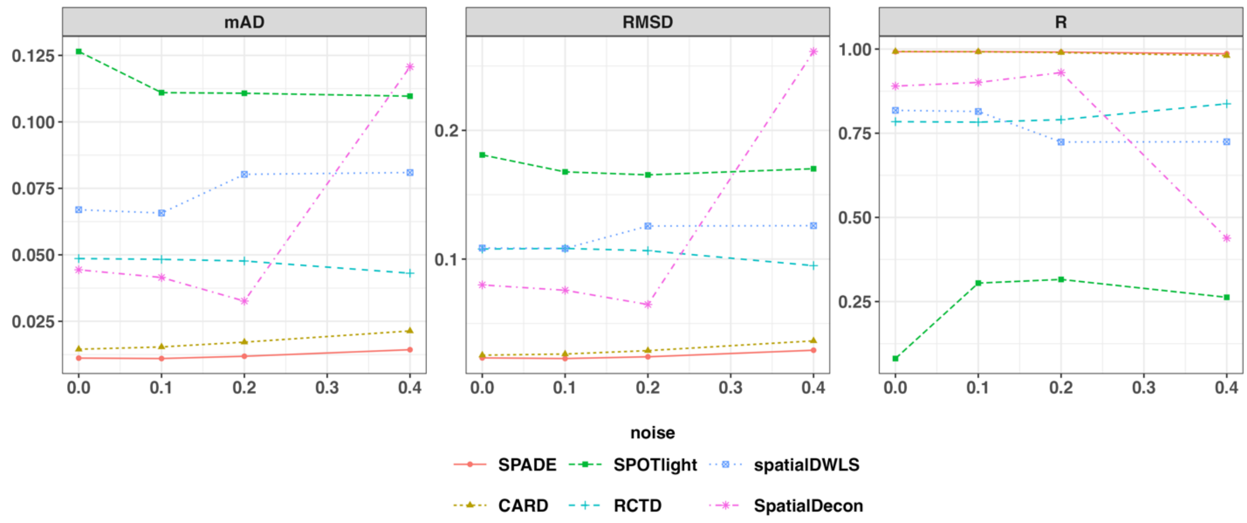

Supplementary Figure 6. Performance comparison at different noise levels in the mouse kidney data. The x-axis represents noise levels, and the y-axis is the values corresponding to each evaluation metric. Each panel illustrates a distinct evaluation outcome. Results obtained from different methods are represented by colored lines. Lower values are desirable for metrics like mAD and RMSD, while a higher value is preferable for the correlation coefficient, denoted as R.

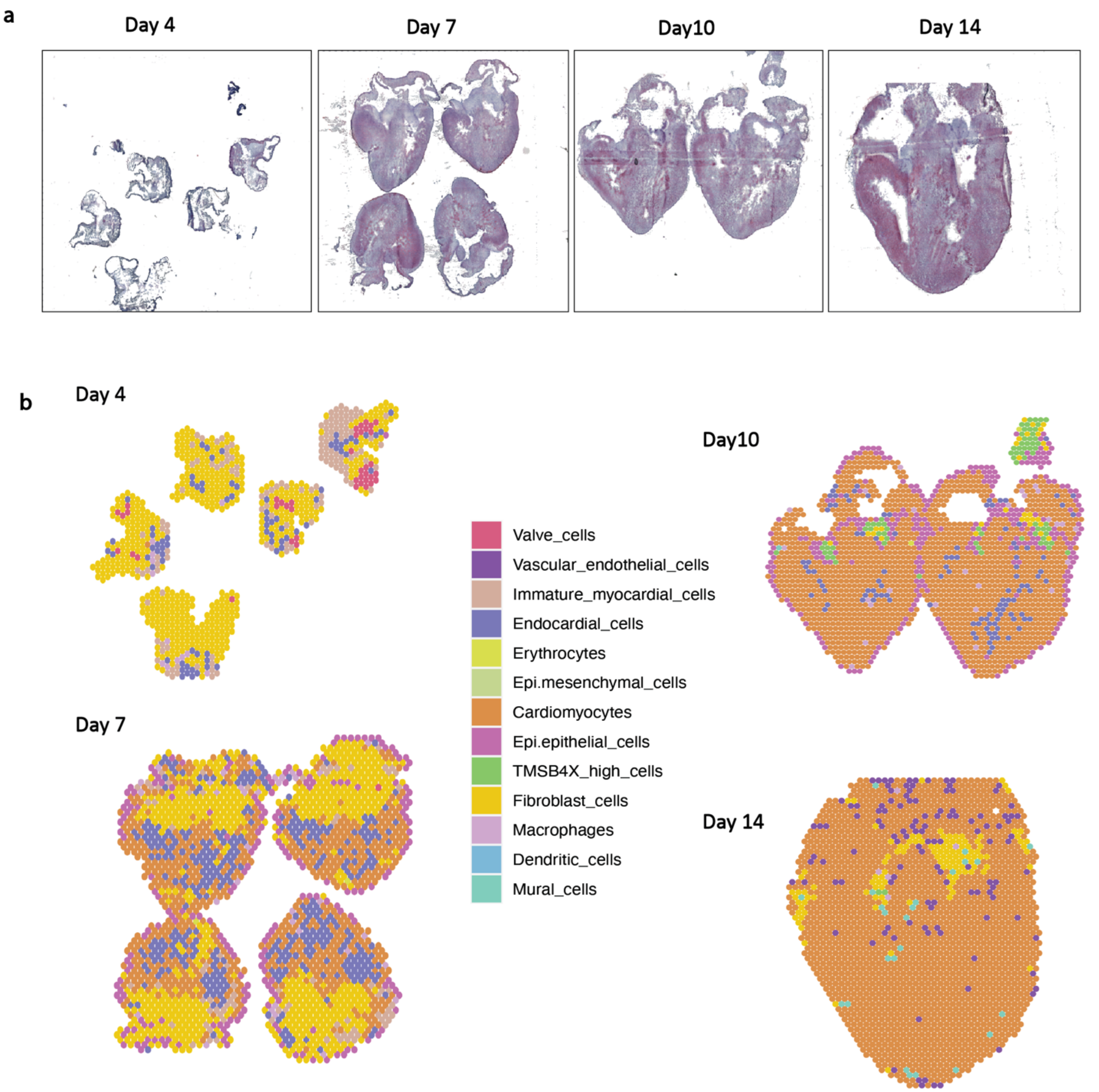

Supplementary Figure 7. Developmental chicken heart. a. H&E staining of the chicken heart at day 4, day 7, day 10 and day 14 from original publication [1]. b. Scatter pie plot for mapping cell type proportion to each location. Each location is represented by a pie plot indicated the cell type composition estimated by SPADE. Color represents cell type. At day 4, most of locations are dominated by immature myocardial cells and fibroblast cells. While at day 14, the cardiomyocyte became the dominant cell type. The trend is consistent with the discussion in result section.

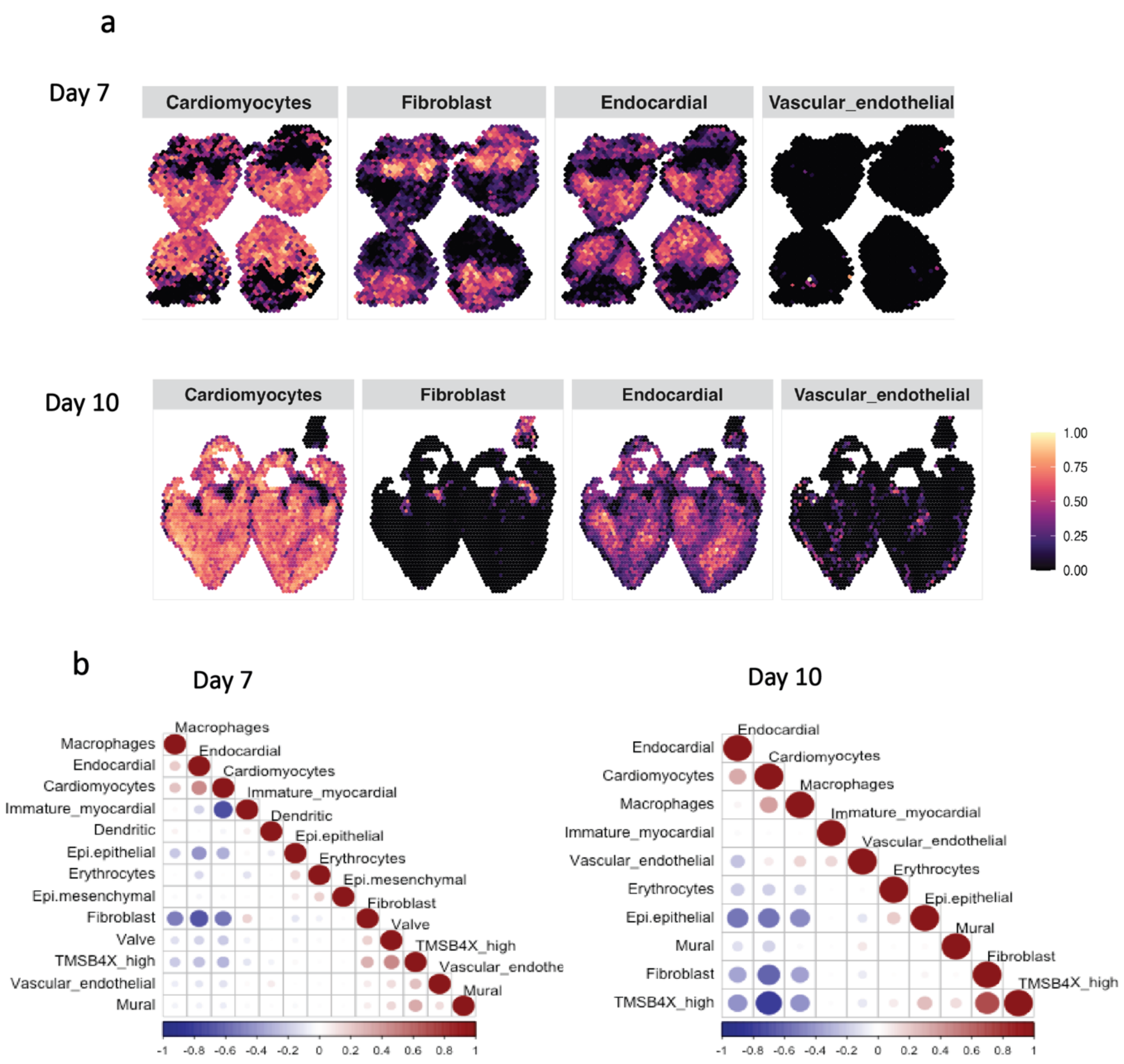

Supplementary Figure 8. Chicken heart at day 7 and day 10. a. Cardiomyocytes, fibroblast, endocardial, vascular endothelial cells proportion in each location at day 7 and day 10. The color represents the level of proportion. It is clear that cardiomyocytes and vascular endothelial cells are increasing the proportion, and fibroblast and endocardial cells are decreasing with the date. b. Cell type colocalization at day 7 and day 10. The correlation between cell types are increasing the development of heart.

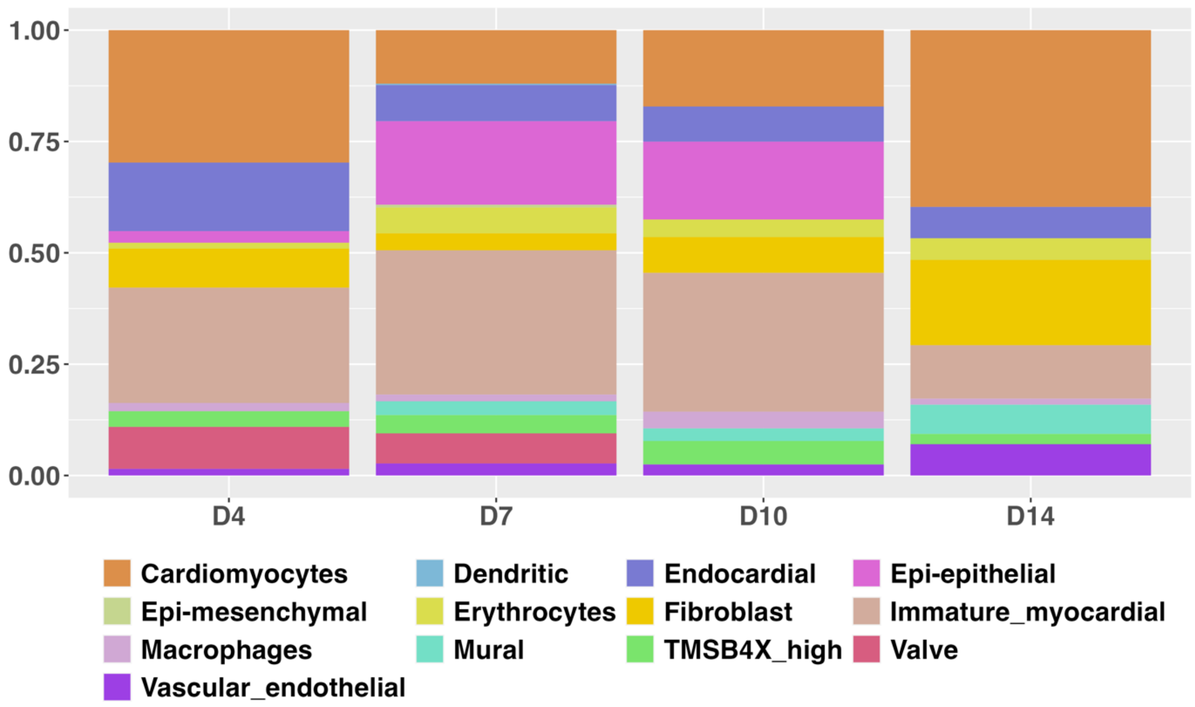

Supplementary Figure 9. Estimated cell type proportion for the Chicken heart data from CARD. colors representing different cell types.

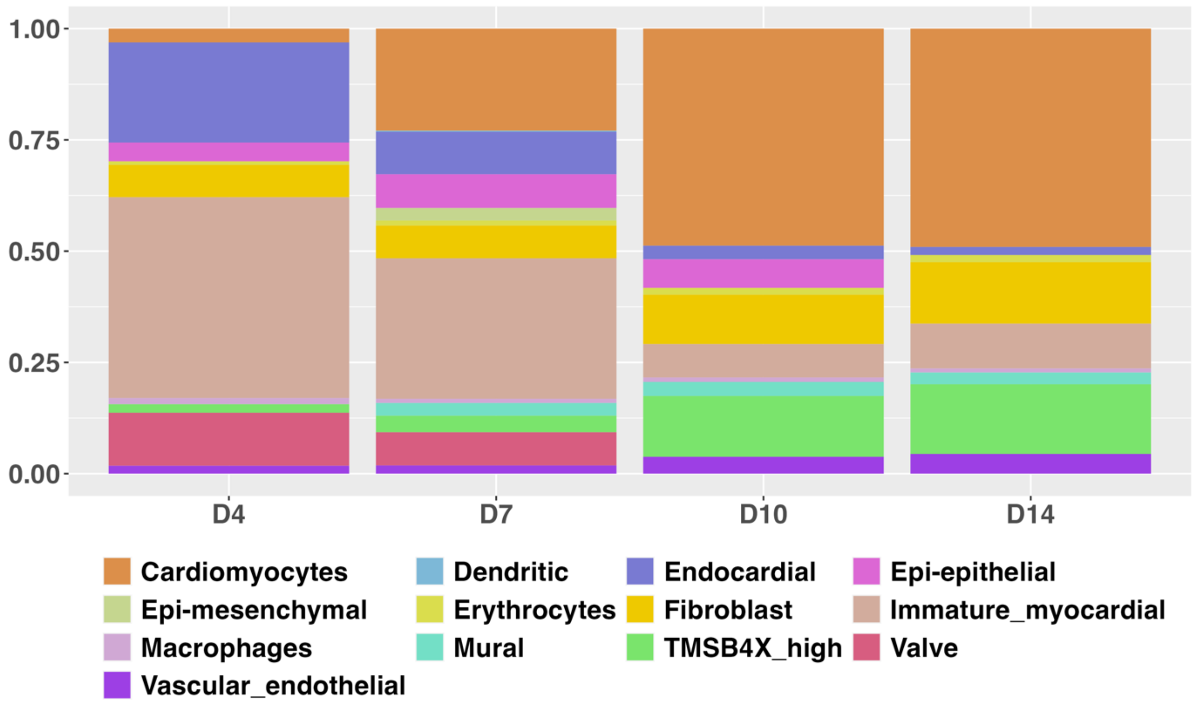

Supplementary Figure 10. Estimated cell type proportion for the Chicken heart data from RCTD. colors representing different cell types.

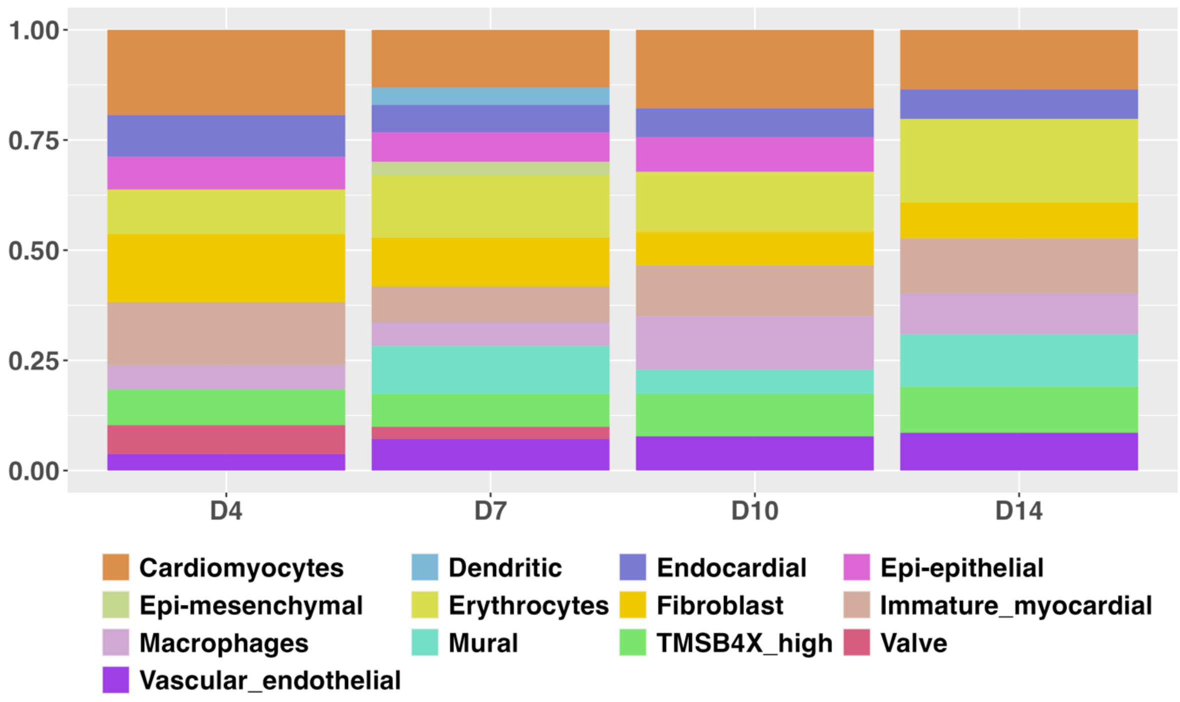

Supplementary Figure 11. Estimated cell type proportion for the Chicken heart data from SPOTlight. colors representing different cell types.

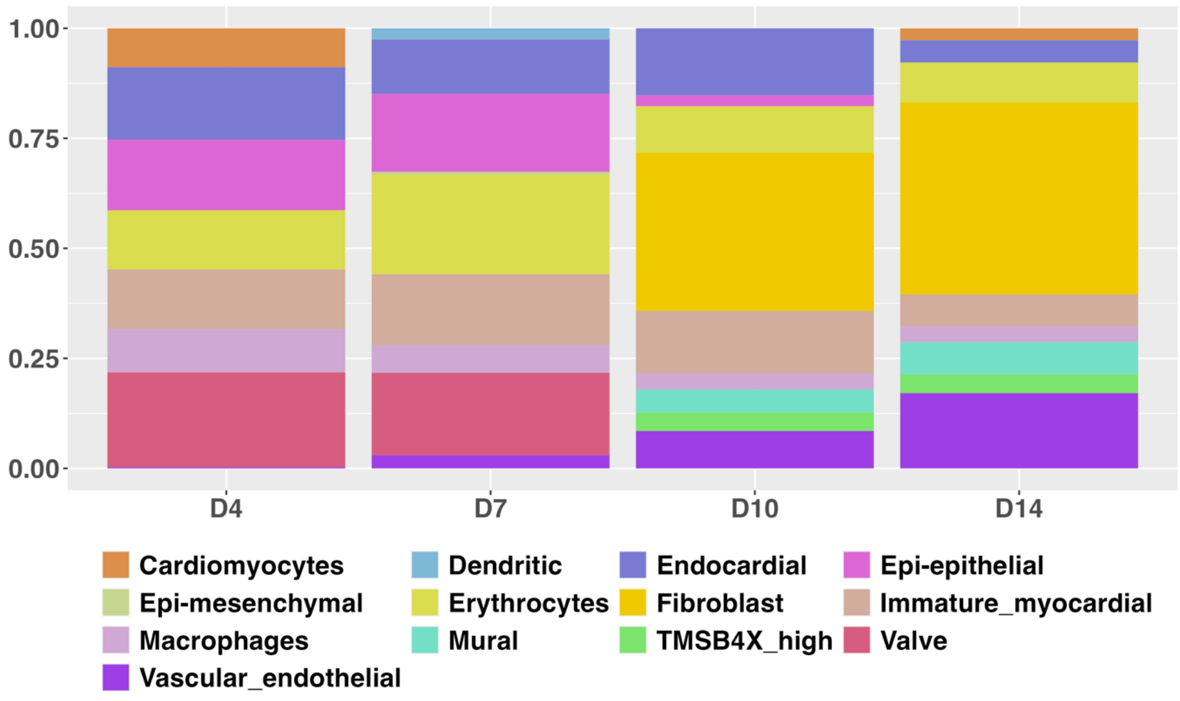

Supplementary Figure 12. Estimated cell type proportion for the Chicken heart data from SpatialDecon. colors representing different cell types.

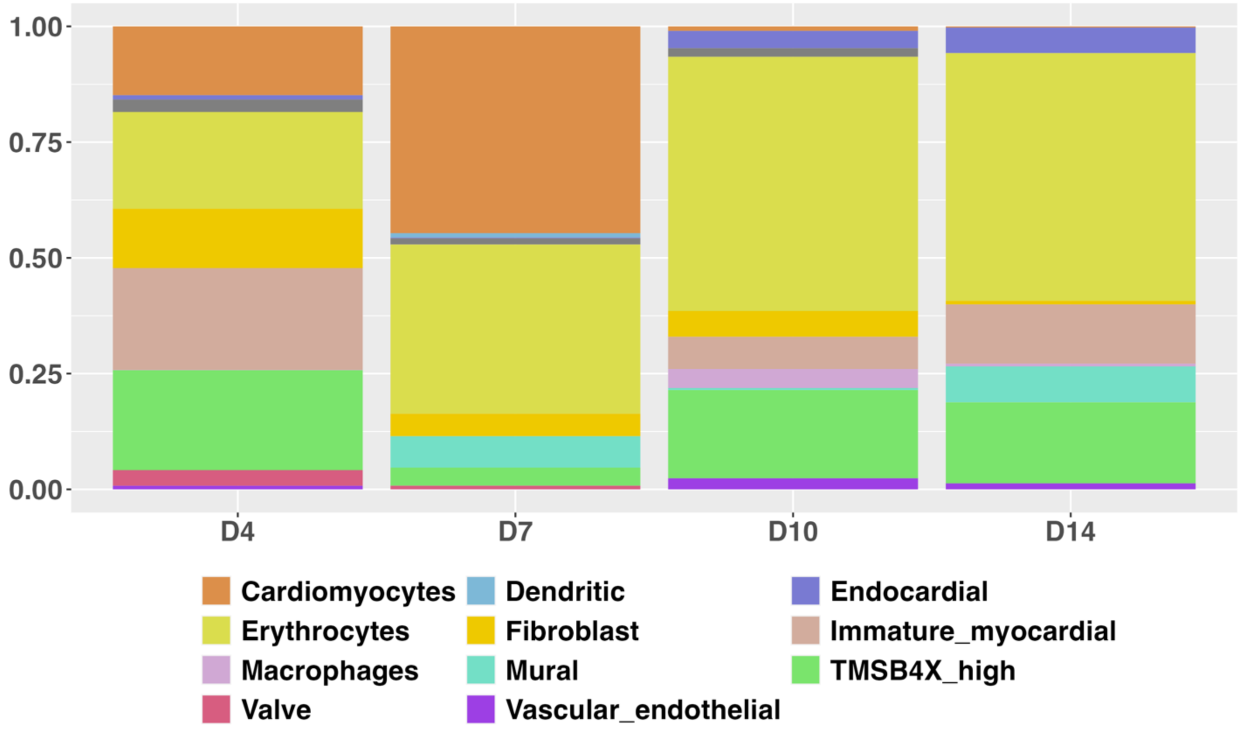

Supplementary Figure 13. Estimated cell type proportion for the Chicken heart data from spatialDWLS. colors representing different cell types.

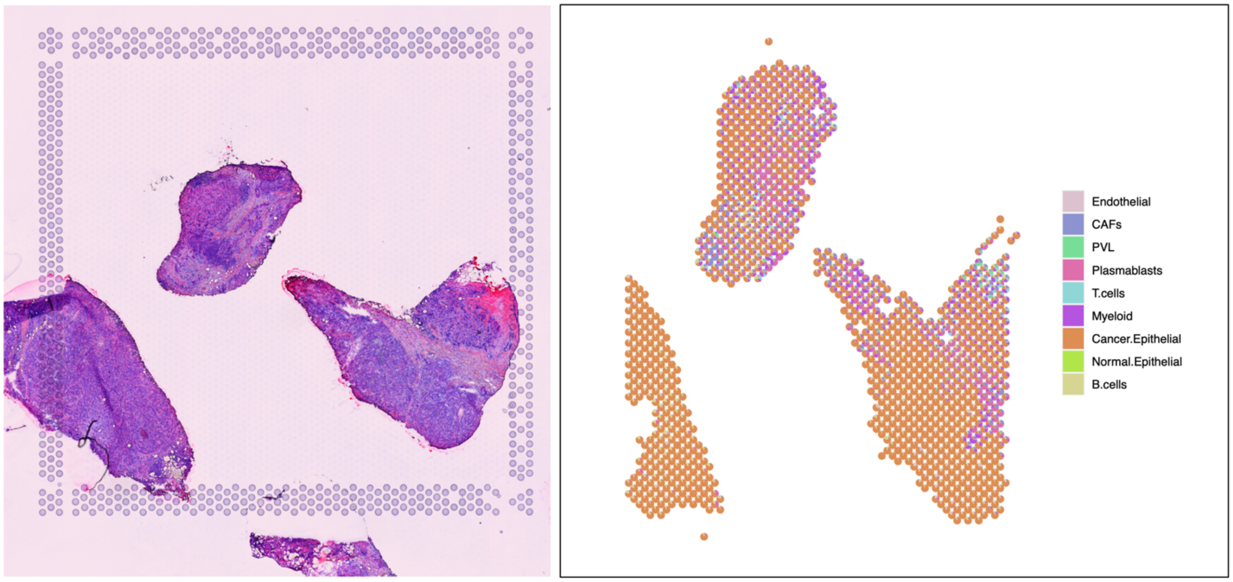

Supplementary Figure 14. Human breast cancer. Left is the H&E staining [2] for breast cancer tissue. Right is the scatter pie plot for estimated cell types at each location. Each location is represented by a pie plot colored by different cell type composition. The cancer epithelial cells are dominated at most of locations, followed by plasmablasts and myeloid cells.

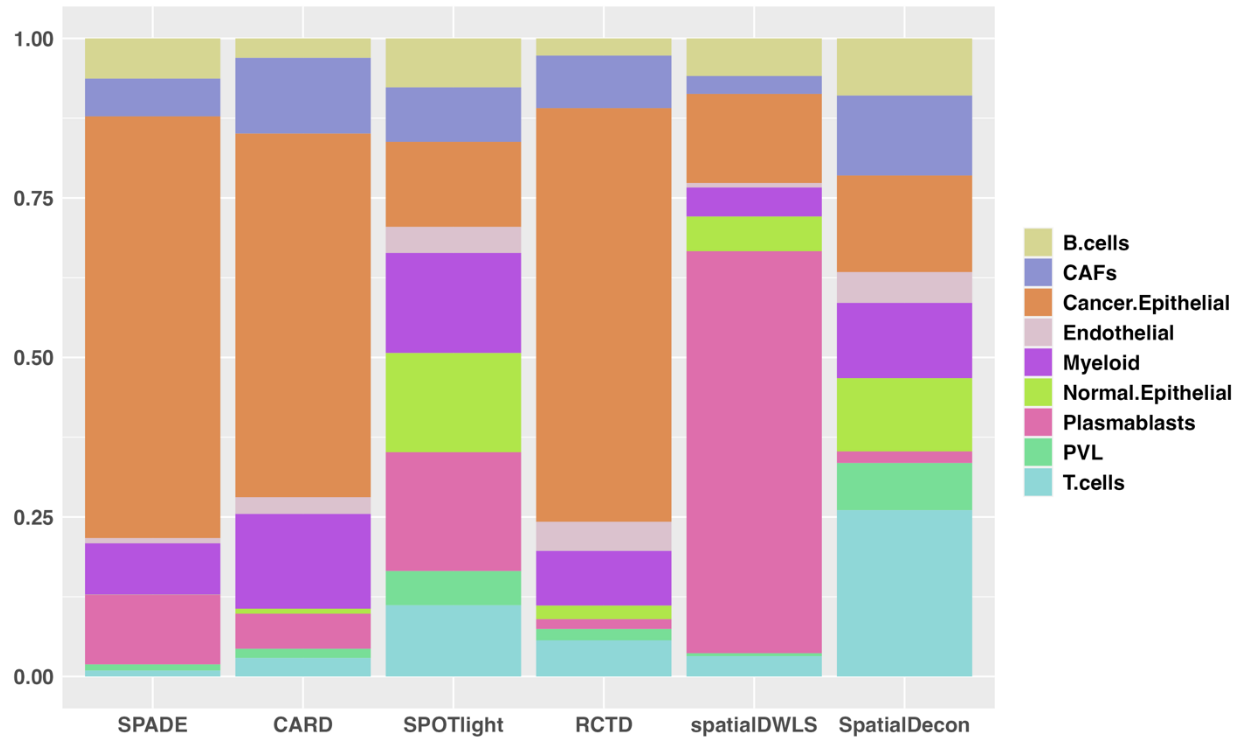

Supplementary Figure 15. Comparison of the estimated cell type proportion for the human breast cancer data. Colors representing different cell types. Each bar represents the proportion estimated from difference methods.

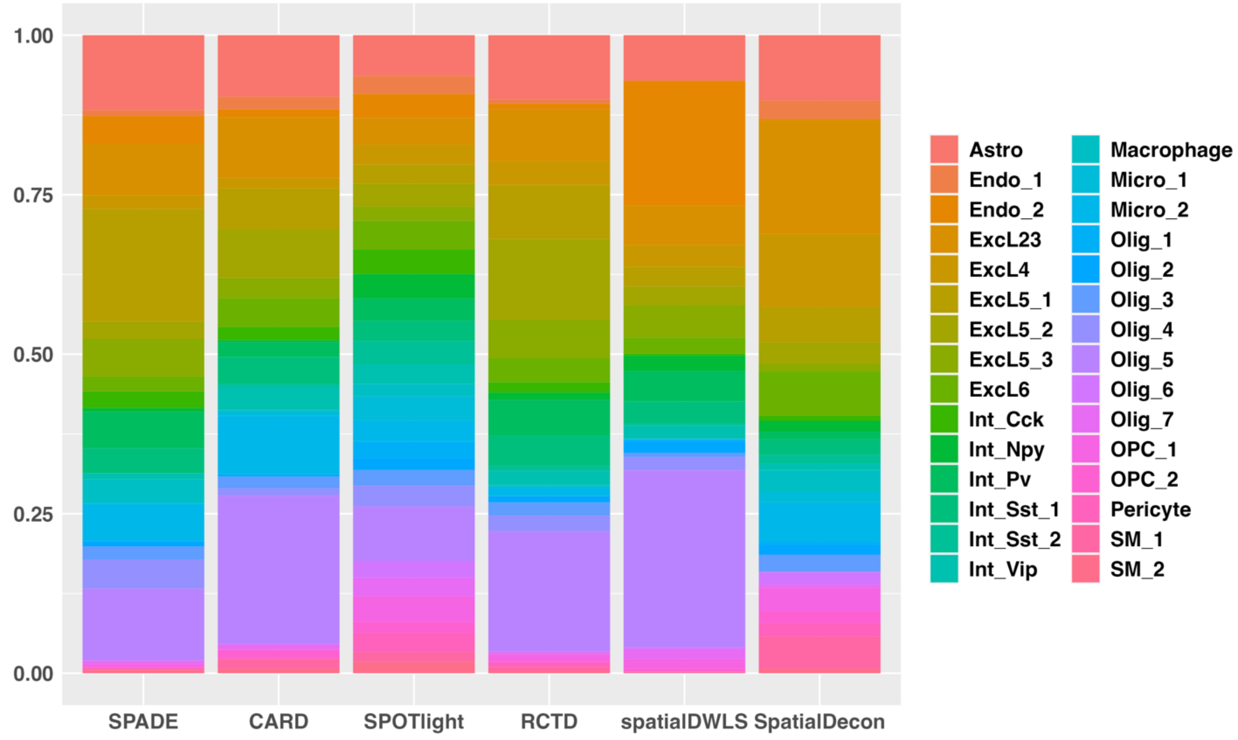

Supplementary Figure 16. Comparison of the estimated cell type proportion for the mouse visual cortex data. Colors representing different cell types. Each bar represents the proportion estimated from difference methods.

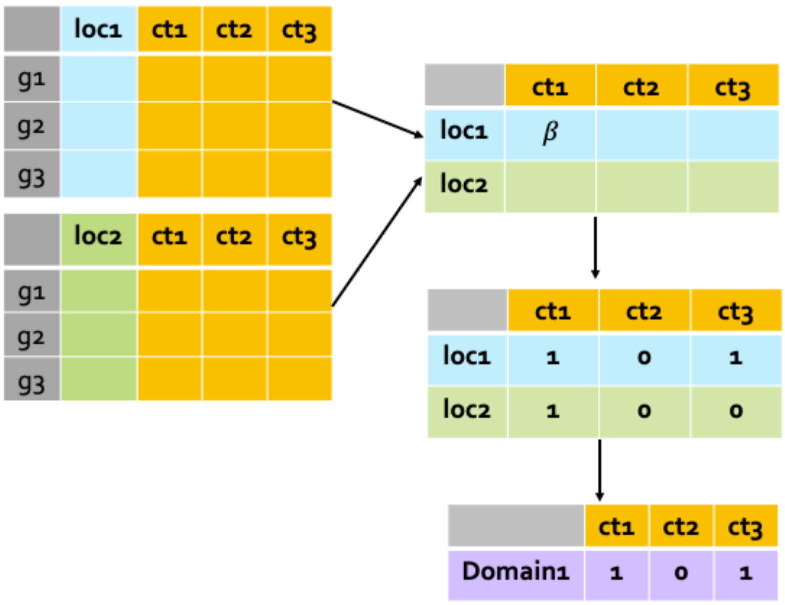

Supplementary Figure 17. Determine number of cell types within each spatial domain by Lasso regression. A gene expression matrix is constructed for each location within every spatial domain, encompassing the expression data for each cell type. Lasso regression is executed to estimate the coefficient of cell type for each location. Following this, for every domain, a coefficient matrix is obtained, denoting the relationship between cell types and locations. To convert the coefficient matrix to a binary matrix, we employ adaptive thresholding. This thresholding method modifies the threshold value for each entry based on the local intensity distribution of the matrix. The R package EBImage's "thresh" function is utilized to implement this approach. Evaluating the binary matrix based on the frequency of 1's determines if a cell type is present within that domain. A value of 1 indicates the existence of a cell type at that location, and if at least one location have a 1 value, then this cell type is considered present in that domain.

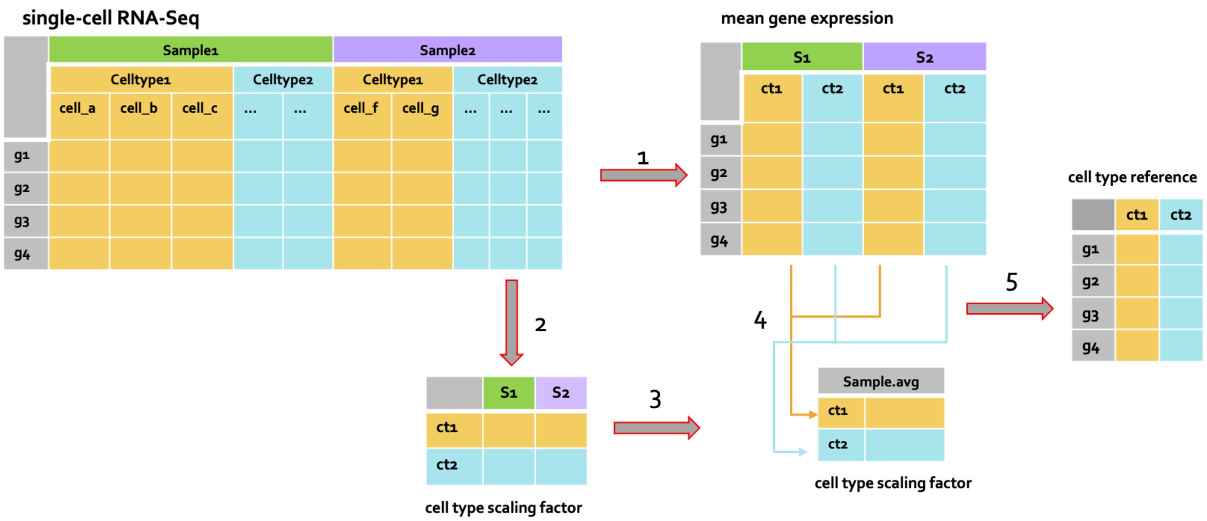

Supplementary Figure 18. Construction reference data. The scRNA-seq data comprises gene expression values for multiple cells that belong to different cell types and samples. To construct a reference for each cell type, we first calculate the average gene expression for each cell type within each sample. This is done by taking each gene expression value over all cells, dividing it by the total gene expression for each cell type and sample. Next, for each cell type, we calculate a scaling factor for each sample by scaling the total gene expression by the number of cells for each cell type. We then average the scaling factor across all samples to get an averaged scaling factor per cell type. Finally, we scale the cell type-specific mean gene expression by its corresponding cell type scaling factor and average it over all samples to obtain sample-corrected cell type-specific gene expression. This can be used as a reference to infer cell type information for cell type deconvolution.

Supplementary References

[1] M. Mantri *et al.*, “Spatiotemporal single-cell RNA sequencing of developing chicken hearts identifies interplay between cellular differentiation and morphogenesis,” *Nat. Commun.*, vol. 12, no. 1, p. 1771, Mar. 2021, doi: 10.1038/s41467-021-21892-z.

[2] S. Z. Wu *et al.*, “A single-cell and spatially resolved atlas of human breast cancers,” *Nat. Genet.*, vol. 53, no. 9, pp. 1334–1347, Sep. 2021, doi: 10.1038/s41588-021-00911-1.
