# Supplementary material for: SPADE: spatial deconvolution for domain specific cell-type estimation
Source: Commun Biol. 2024 Apr 17;7:469. doi: 10.1038/s42003-024-06172-y (PMC11024133; doi:10.1038/s42003-024-06172-y)
Supplement: Supplementary file 3 — Description of Supplementary Materials [file 42003_2024_6172_MOESM3_ESM.docx]

**Description of Additional Supplementary Files**

**File name:** Supplementary Data 1

**Description:** Source dataset for Figure 2

**File name:** Supplementary Data 2

**Description:** Source dataset for Figure 3

**File name:** Supplementary Data 3

**Description:** Source dataset for Figure 4

**File name:** Supplementary Data 4

**Description:** Source dataset for Figure 5

**File name:** Supplementary Data 5

**Description:** Source dataset for Figure 6
